# The economic burden of measles in children under five in Bangladesh

**DOI:** 10.1186/s12913-020-05880-5

**Published:** 2020-11-10

**Authors:** Gatien de Broucker, Sayem Ahmed, Md. Zahid Hasan, Gazi Golam Mehdi, Jorge Martin Del Campo, Md. Wazed Ali, Md. Jasim Uddin, Dagna Constenla, Bryan Patenaude

**Affiliations:** 1 International Vaccine Access Center, Department of International Health, Johns Hopkins Bloomberg School of Public Health, 415 North Washington Street, #530, Baltimore, MD 21231 USA; 2 International Centre for Diarrhoeal Disease Research, Dhaka, Bangladesh; 3 Liverpool School of Tropical Disease (LSTM), Liverpool, UK; 4 GlaxoSmithKline Plc, Panama City, Panama

**Keywords:** Economic burden, Cost of illness, Measles, Bangladesh, Caregiver cost

## Abstract

**Background:**

This study estimated the economic cost of treating measles in children under-5 in Bangladesh from the caregiver, government, and societal perspectives.

**Method:**

We conducted an incidence-based study using an ingredient-based approach. We surveyed the administrative staff and the healthcare professionals at the facilities, recording their estimates supported by administrative data from the healthcare perspective. We conducted 100 face-to-face caregiver interviews at discharge and phone interviews 7 to 14 days post-discharge to capture all expenses, including time costs related to measles. All costs are in 2018 USD ($).

**Results:**

From a societal perspective, a hospitalized and ambulatory case of measles cost $159 and $18, respectively. On average, the government spent $22 per hospitalized case of measles. At the same time, caregivers incurred $131 and $182 in economic costs, including $48 and $83 in out-of-pocket expenses in public and private not-for-profit facilities, respectively. Seventy-eight percent of the poorest caregivers faced catastrophic health expenditures compared to 21% of the richest. In 2018, 2263 cases of measles were confirmed, totaling $348,073 in economic costs to Bangladeshi society, with $121,842 in out-of-pocket payments for households.

**Conclusion:**

The resurgence of measles outbreaks is a substantial cost for society, requiring significant short-term public expenditures, putting households into a precarious financial situation. Improving vaccination coverage in areas where it is deficient (Sylhet division in our study) would likely alleviate most of this burden.

**Supplementary Information:**

The online version contains supplementary material available at 10.1186/s12913-020-05880-5.

## Background

The vaccination coverage for measles worldwide is estimated at 86% for the first dose of measles-containing vaccine (MCV1) and 69% for MCV2 in 2018 [1], leaving most of the measles cases to occur as outbreaks driven by gaps in vaccination coverage and humanitarian crises [2]. In 2005, Bangladesh carried out a mass measles vaccination catch-up campaign, in addition to its routine immunization, effectively bringing vaccination coverage for MCV1 in the 89–97% range nationally for the last ten years [1]. Measles vaccination effectively curbed the incidence of measles to the point where the Government of Bangladesh enacted a strategy for measles elimination in 2014 as they added a second dose (MCV2) to their expanded program for immunization (EPI) in 2012 [3]. Including the cost of the vaccine (Gavi pricing applied) and its delivery, measles vaccination in Bangladesh from 2011 to 2030 is estimated to reach about $136,116,356, which translates to $1.33 per dose on average [4].

The reported vaccination coverage remains high nationally: 92 and 83% for the first and second dose of MCV [1]. Nevertheless, the country is experiencing a resurgence of measles cases with over 1069 and 4001 confirmed cases in 2016 and 2017, respectively, compared to less than 400 from 2013 to 15 [5]. The increased prevalence of measles cases was driven in part from the South-East of the country with the Rohingya migration in Chittagong district [6] and by differences in vaccination coverage across divisions [7].

The value of vaccines with a longstanding presence in national EPI is poorly understood in economic terms. However, with declining stockpiles (yellow fever), the discovery of gaps in immunity in young adults (rubella) [8] and a sharp recrudescence of cases (measles), strategies to sustain and improve the coverage of these existing vaccines are at the forefront, potentially delaying the introduction of vaccines against other diseases. To our knowledge, there is no other empirical cost-of-illness study for measles in low-income settings: only a few were done in lower- and upper-middle-income countries [9–11].

We aimed to fill this gap with measles cost estimates for Bangladesh, including inpatient and outpatient costs from the different sectors providing care. This study was part of an extensive stand-alone cost-of-illness study producing estimates of the cost of measles, pneumonia, and diarrhea for the healthcare facility, caregiver, and society in Bangladesh.

## Methods

### Study design

The study focused on costing an episode of measles, using the same study design (epidemiological and costing approaches) that we used to assess the economic burden of measles in Uganda [12]. We used an ingredient-based approach, where the cost and quantity of individual items (e.g., medications, supplies) that were reportedly used to care for this episode were assessed and aggregated. This approach allows us to estimate the “real-world” cost [13] of an episode of measles, potentially including medications and items not typically recommended in treatment guidelines. Further explanations of these economic approaches can be found in Vassall et al. and Jo [13, 14].

### Study population and sites

**Fig. 1:**
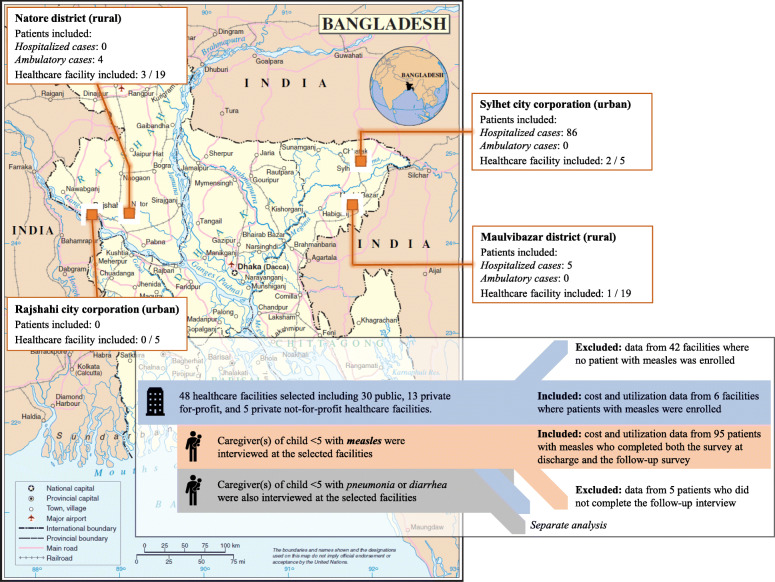
Map of the study sites. Map based on the United Nations Map No. 3711 Rev. 2, January 2004

The study was focused on two divisions with different levels of vaccination coverage: Sylhet (low coverage at 61.1%) and Rajshahi (high coverage at 83.6%) [15]. In each division, we selected a district (Maulvibazar and Natore districts) and a city corporation (Sylhet and Rajshahi city corporations), representative of rural and urban settings. We included 24 healthcare facilities in each division (19 in city corporations and 5 in rural districts, see Fig. 1), selected based on the number of measles, pneumonia, and diarrhea cases reported for the prior year (2015–16) and to represent different facility levels and sectors: we included 30 public and 18 private for-profit and not-for-profit facilities. Note that only six facilities presented measles cases by the end of data collection: only the costs from those facilities were included in our analysis (listed in Table 1). We collected additional information on the costs of medications from 20 pharmacies from the area surrounding the facilities – selected on the recommendation of the healthcare facility staff. Pharmacies were all privately owned and registered.

**Table 1:** Government costs for an episode of measles in 2018 US dollars ($)

Level, name & type of care	n	Service costs					
		Capital	Overhead	Labor	Supplies	Medications^a^	Total
**Primary level**							
Bashbaria Community Clinic							
*Outpatient care*	1	$0.46	$0.00	$0.54	$0.00	$0.00	**$1.00**
Baraigram Upazila Health Complex							
*Outpatient care*	2	$0.08	$0.13	$0.99	$0.02	$0.49	**$1.71**
Zoary Union Sub Center							
*Outpatient care*	1	$0.23	$0.00	$1.20	$0.00	$0.00	**$1.43**
**Secondary level**							
Moulvibazar District Hospital, Moulvibazar							
*Inpatient care*	5	$0.32	$0.98	$5.76	$0.21	$10.30	**$17.56**
**Tertiary level**							
Sylhet MAG Osmani Medical College Hospital							
*Inpatient care*	64	$1.32	$0.81	$10.29	$0.92	$9.53	**$22.88**

Notes: n, number of caregivers interviewed at the facility. ^a^Medications provided included: paracetamol, antihistamine, vitamin A, calamine lotion, oral analgesic gel, and chloramphenicol eye drop

The adult caregivers of children 0–59 months of age with a diagnosis at the time of facility discharge of (suspected) measles were interviewed. Measles diagnosis was based on clinical assessment and was not always confirmed by laboratory tests. We assumed that: (1) healthcare professionals were likely to provide an accurate assessment of measles in locations where outbreaks occur regularly as this is the case for Sylhet, and (2) the treatment provided corresponded to a treatment for measles. We did not include cases that reported only a rash without specifying “measles” in the patients’ medical records. We excluded cases also diagnosed with other diseases (e.g., HIV, pneumonia).

### Data collection

The surveys were administered in Bengali on tablets using KoBoToolbox (Cambridge, MA), open-source software used to collect, manage, and analyze data in challenging settings. From August 2017 to May 2018, a team of six field research assistants interviewed the staff from the 48 selected healthcare facilities to collect data on healthcare facility costs and utilization. We triangulated our data with administrative records. Healthcare facility costs included capital costs (infrastructure, furniture, and medical equipment), overhead costs, labor costs (staff salaries and benefits), medical supplies, and medications used for diagnostic tests, hospitalization, and treatment. Data collection was limited to the pediatric ward in hospitals and medical colleges healthcare facilities. Additional data on medication pricing was retrieved from pharmacies in the private sector and surrounding the selected healthcare facilities to complement missing information from the healthcare facility surveys. The data, questionnaires, and codebooks are available in open access [16].

Caregivers were interviewed at the time of discharge from the facility and 7 to 14 days post-discharge. We collected information about the medical costs, non-medical costs, indirect costs, and time spent in healthcare due to this episode, as well as information about the caregivers’ household: its daily expenditures and its income to assess its socioeconomic status [12].

Cost categories were defined based on the Global Health Cost Consortium (GHCC) and Jo [13, 14], and can be found in detail in the Supplementary Material: Table S1. Costs were collected in Bangladeshi Takas (BDT) and converted to 2018 US dollars, using the following conversion rate: 1 USD = 83.5 BDT [17]. Costs in 2018 BDT are available in the Supplementary Material: Tables S5, S6, S7 and Fig. S1.

### Government costs

From the government perspective, all costs were patient-specific except for overhead, labor, and capital costs since those costs were shared with all other patients. Capital costs were annualized based on a standard lifetime of 50 years for infrastructure and five years for medical equipment with a discount rate of 3% [18, 19]. We faced the same difficulties to apportion overhead, labor and capital costs to measles treatment in Bangladesh as we did in Uganda [12] and used the same methodology to estimate it (see Eq. 1).

1 $$ S=\sum \limits_{\begin{array}{l}i=o\\ {}j=o\end{array}}^{\begin{array}{l}n\\ {}m\end{array}}\frac{cj\times {los}_{i,j}}{P_j} $$

Where *S* is the total cost of overhead, labor, and capital attributable to an episode of measles per facility, *c*_*j*_ the total annual cost, *p*_*j*_ the annual number of patients who used the facility and with *los*_*i,j*_ the length of stay in days for patient *i* over *n* total patients whose caregiver was interviewed, and for healthcare facility *j* over *m* total facilities.

### Caregiver costs

Out-of-pocket payments included hospital fees, medications, medical supplies, transportation, and meals. Adding indirect costs, we calculated the total economic cost for caregivers. Differences in direct, indirect and overall costs were assessed (see Supplementary Material: Table S2) and discussed in the Results.

### Catastrophic health expenditures and household socioeconomic status

We examined the proportion of the monthly income of the head of the household and the monthly household expenditures spent on out-of-pocket payments. Monthly expenditures included food, clothing, supplies, leisure, tax paid, other healthcare expenses, and other expenses. We determined that a household was experiencing catastrophic health expenditures related to this measles episode when they paid in out-of-pocket payments over 10% of their income, 10% or 25% of their monthly expenditures or 40% of their monthly expenditures without food [20, 21].

Each household’s socioeconomic status was defined based on asset scores generated through a principle component analysis (PCA) approach, grouped into asset quintiles [12, 22] {Filmer, 2001 #4}. In the first quintile (poorest households), 17 households obtain the same score, thus explaining the unequal proportion between the first and second quintiles.

## Results

We captured a total of 100 measles cases during the data collection period; however, 5 cases were excluded because the caregivers associated with these cases did not complete the post-discharge interview. Most measles cases were captured in public healthcare facilities. Sixty-four hospitalized cases in Sylhet MAG Osmani Medical College Hospital (tertiary level) in Sylhet City Corporation, and 5 in Maulvibazar District Hospital (secondary) in Maulvibazar district (both in Sylhet division). Four outpatient cases were also captured primary level facilities in Natore district (Rajshahi division): 2 in Baraigram Upazila Health Complex, 1 in Bashbaria Community Clinic, and 1 in Zoary Union Sub Center. Twenty-two hospitalized cases came from Lions Child Hospital (secondary level), a private not-for-profit hospital in Sylhet City Corporation.

In our sample, 58% of the children affected were aged under one year, with more girls (59%) than boys. Most of them came from rural areas (88%) to get treated in hospitals located in urban settings (95% of all admissions). Most children hospitalized due to their measles episode stayed five days or more in healthcare facilities (71%), with no significant difference in length of stay between public and private facilities.

### Cost-of-illness estimates

Government facilities spent an average of $1.95 per outpatient case and $21.59 for a hospitalized case. Costs for outpatient cases ranged from $1.00 to $1.71, mainly driven by the estimated labor (54–84%) and capital costs (5–46%). Hospitalized cases in Maulvibazar District Hospital costed an average of $17.56, and those in Sylhet MAG Osmani Medical College Hospital an average of $22.88, both driven by labor (33 and 45%) and medication costs (59 and 42%). Overhead costs were also significant in most facilities at 4–8%. Table 1 presents detailed cost estimates from the government perspective.

Overall, the mean economic cost per episode of measles for caregivers was $138, with an average out-of-pocket cost of $54. Table 2 presents the estimates by type of cost. Caregivers who used public healthcare facilities spent significantly less than those who used private not-for-profit facilities for a hospitalized measles (*p* ≤ 0.01, see Supplementary Material: Table S2). On average, caregivers spent a total of $131, including $48 in out-of-pocket expenses in the public sector compared to $182 in total costs and $83 in out-of-pocket in the private not-for-profit facility. Caregivers’ time spent in healthcare facilities for inpatient care was nearly the same between public and private facilities with 4.5 days and 4.4 days, valued at $83 and $100, respectively. Neither the time spent on healthcare nor the indirect costs were significantly different. Expectedly, longer lengths of stay (5 days or more) meant significantly higher direct costs, indirect and overall costs.

**Table 2:** Total caregiver costs for a hospitalized episode of measles in 2018 US dollars ($), and time loss in days

**Timing**	**Cost**	**INPATIENT VISIT**									
		**Public healthcare facilities (*n*** **= 69)**					**Private not-for-profit healthcare facilities (*n*** **= 22)**				
		Mean	SD	95% CI		n(c > 0)	Mean	SD	95% CI		n(c > 0)
**Before current visit**^**a**^	Direct medical	$3.53	$6.78	$1.90	$5.16	31	$7.17	$6.74	$4.18	$10.16	17
	Direct non-medical	$0.36	$1.60	-$0.02	$0.75	4	$1.52	$3.43	$0.00	$3.04	5
	Indirect	$28.85	$30.02	$21.58	$36.12	69	$24.72	$23.02	$14.51	$34.92	22
	*Time loss [days]*	1.6	1.2	1.3	1.9	69	1.1	0.7	0.8	1.4	22
**Current visit**	Direct medical	$14.65	$10.19	$12.19	$17.09	69	$42.42	$21.17	$33.03	$51.81	22
	Direct non-medical	$26.00	$13.71	$22.69	$29.29	69	$28.32	$14.74	$21.78	$34.86	22
	Indirect	$54.51	$42.81	$44.23	$64.80	69	$74.86	$55.25	$50.37	$99.37	22
	*Time loss [days]*	2.9	1.4	2.5	3.2	69	3.3	0.9	2.8	3.7	22
**Follow-up**^**a**^	Direct medical	$2.57	$2.90	$1.88	$3.27	52	$3.07	$2.99	$1.75	$4.40	16
	Direct non-medical	$0.54	$4.32	-$0.50	$1.57	2	$0.11	$0.30	-$0.02	$0.24	3
	Indirect	$0.44	$3.60	-$0.43	$1.31	2	$0.00	$0.00	$0.00	$0.00	0
	*Time loss [days]*	0.0	0.3	0.0	0.1	2	0.0	0.0	0.0	0.0	0
**Total out-of-pocket payment**		**$47.64**	**$21.87**	**$44.73**	**$55.43**	**69**	**$82.61**	**$34.92**	**$70.02**	**$101.32**	**22**
**Total economic cost**		**$131.03**	**$83.65**	**$110.93**	**$151.13**	**69**	**$182.20**	**$96.59**	**$139.38**	**$225.02**	**22**
**Timing**	**Cost**	**OUTPATIENT VISIT**					**INPATIENT & OUTPATIENT VISITS**				
		**Public healthcare facilities (*n*** **= 4)**					**All healthcare facilities (*n*** **= 95)**				
		Mean	SD	95% CI		n(c > 0)	Mean	SD	95% CI		n(c > 0)
**Before current visit**^**a**^	Direct medical	$0.00	$0.00	$0.00	$0.00	0	$4.23	$6.82	$2.84	$5.62	48
	Direct non-medical	$0.00	$0.00	$0.00	$0.00	0	$0.62	$2.18	$0.17	$1.06	9
	Indirect	$0.29	$0.31	-$0.20	$0.78	4	$26.67	$28.34	$20.86	$32.47	95
	*Time loss [days]*	0.0	0.0	0.0	0.1	4	1.4	1.2	1.2	1.6	95
**Current visit**	Direct medical	$0.02	$0.02	$0.00	$0.04	3	$20.46	$18.19	$16.75	$24.17	94
	Direct non-medical	$0.06	$0.06	-$0.13	$0.25	1	$25.44	$14.64	$22.46	$28.42	92
	Indirect	$13.32	$19.54	-$17.80	$44.42	4	$57.49	$46.69	$47.98	$67.00	95
	*Time loss [days]*	1.1	1.6	−1.5	3.7	4	2.9	1.4	2.6	3.2	95
**Follow-up**^**a**^	Direct medical	$2.08	$2.42	-$1.77	$5.93	2	$2.67	$2.89	$2.08	$3.26	70
	Direct non-medical	$0.36	$0.72	-$0.78	$1.50	1	$0.43	$3.69	-$0.32	$1.18	6
	Indirect	$0.13	$0.26	-$0.29	$0.55	1	$0.32	$3.07	-$0.30	$0.95	3
	*Time loss [days]*	0.0	0.0	0.0	0.0	1	0.0	0.3	−0.0	0.1	3
**Total out-of-pocket payment**		**$2.51**	**$2.89**	**-$3.62**	**$12.22**	**4**	$53.84	$30.87	$47.55	$60.13	**95**
**Total economic cost**		**$16.25**	**$21.37**	**-$17.75**	**$50.25**	**4**	$138.04	$91.02	$119.50	$156.59	**95**

Notes: SD, standard deviation; n(c > 0), number of caregivers with a cost/time spent valued over zero. ^a^ Includes costs incurred at public and private healthcare facilities and providers

For the few outpatient episodes captured (*n* = 4), all in the public healthcare system, caregivers spent an average total cost of $16 with $3 in out-of-pocket expenses – significantly lower than the cost of inpatient cases.

Over the continuum of care for measles, either with hospitalization or ambulatory care, the current visit cost influenced most the total cost: 73 and 82% of the total cost in public – inpatient and outpatient, respectively – and 80% in the private not-for-profit facility. Indirect costs due to productivity loss were the most significant share of the total cost in all facilities and types of care, ranging from 55 to 85%. Caregivers residing in urban areas had statistically significantly lower direct costs with $36 than those in rural areas with $56 (*p* = 0.04). There was no difference in costs between male and female children and caregivers.

When grouping the caregivers by asset quintiles, we found that the only significant difference lay in the indirect costs for inpatient care of the 5th quintile compared to the 1st, 2nd, and 3rd quintiles (*p* < 0.05). Caregivers in the poorer three quintiles incurred direct costs from $39 to $70, whereas caregivers in the wealthiest quintile incurred costs of $138. This difference in indirect costs was driven by differences in income, as there was no significant difference in time loss between quintiles. Additionally, all outpatient cases happened in the 2nd quintile, making its associated costs significantly lower than in the other quintiles.

### Economic burden

The economic cost of an episode of measles contributed to 8% of the annual national gross domestic product (GDP) per capita. Based on our sample, 89% of the caregivers reported having spent over 10% of their monthly household income on treating measles. This cost represented, on average, 32% of their monthly income.

**Fig. 2:**
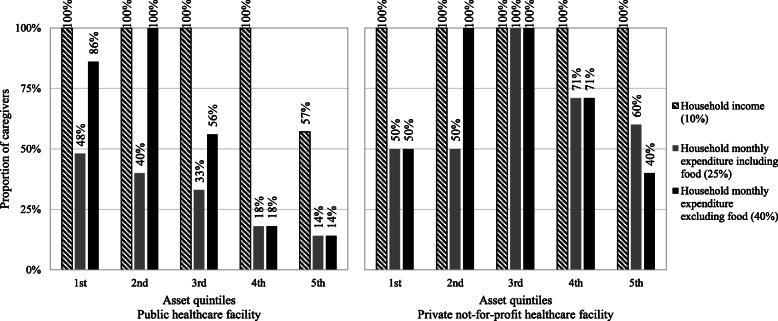
Proportion of caregivers facing catastrophic health expenditures for inpatient care by asset quintile in Bangladesh. Note: The four outpatient cases were excluded from the chart

Focusing on household consumption, 92% of caregivers reported that a measles episode accounted for 10% of their household expenditures, and 44% spending over 25% of their household expenditure. When excluding food, 57% reported spending over 40% of their household expenditure. The proportion of households experiencing catastrophic health expenditures decreased with richer asset quintiles: focusing on daily consumption excluding food, 78% of caregivers in the poorest 1st asset quintile faced catastrophic health expenditures, compared to 60, 49 and 21% in the 3rd, 4th and 5th quintiles, respectively. As shown in Fig. 2, both users of the public and private facilities significantly incur catastrophic health expenditures.

Ninety percent of caregivers reported coping with the additional expenses by using their savings, in combination with getting a loan from a bank or a lending institution (46%) or borrowing from friends (2%). Caregivers in the richest 4th and 5th quintiles all reported relying primarily on savings to fund measles-related treatment, with several (33 and 5%, respectively) who reported having to take a loan. In the poorest 1st and 2nd quintiles, the inverse trend was observed with 81 and 43%, respectively, using their savings, and with 81 and 71% having to take a loan to pay for a measles episode.

### Societal costs and country-level cost of measles

**Fig. 3:**
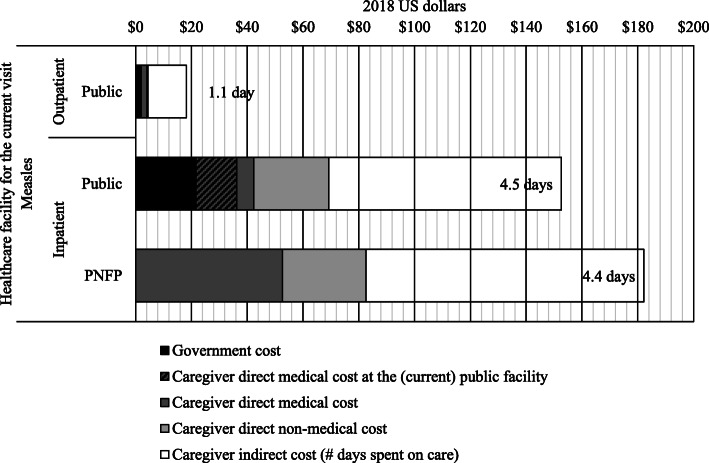
Societal costs for measles in Bangladesh. PNFP, Private not-for-profit sector

From a societal perspective, a hospitalized episode of measles costed $159 and an outpatient episode $18. Across sectors, the societal cost of a hospitalized measles was $153 when using public healthcare and $182 when accessing private not-for-profit healthcare. Direct medical costs contributed to 27 and 29% of the cost for users of public and private facilities, respectively, albeit a different distribution: half of the cost was borne by the government in public facilities (see Fig. 3). Non-medical costs, including transportation and meals, took 18% of the societal cost for those using public facilities and 16% for private facilities, indicating that the caregiver made significant out-of-pocket payments to access healthcare. Indirect costs took the largest share of the societal cost, with 55% at public and private facilities.

By the end of 2018, when data collection was completed, there were 2263 confirmed cases of measles [5], causing $348,073 in societal cost, including $121,842 for Bangladeshi households to bear in out-of-pocket expenses. Suppose we include the previous two years when the incidence rate for measles spiked and used our 2018 average cost. In that case, we estimate the societal, economic cost at $1,127,892, with $394,815 in caregiver out-of-pocket expenses for 2016–2018.

## Discussion

Government provision of healthcare services significantly alleviates the economic burden of measles from households. Nevertheless, there is still room for progress, working towards universal health coverage.

First, while a significant share of the medical costs ($20) was effectively shifted from the caregivers to the government, caregivers still needed to cover $21 (including $6 spent before and after, see Fig. 3). All patients reported paying fees for the visit in public facilities, and most reported paying for medications (97%) and laboratory tests and medical investigations (55%) (see Supplementary Material: Table S6). The remaining medical expense in public healthcare facilities, added to non-medical costs, is enough to hurt the household’s financial security: most of the poorest caregivers seeking healthcare in public facilities still face catastrophic health expenditures (see Fig. 2). Eliminating user fees and reducing the cost of medications for caregivers at public healthcare facilities is a clear step towards universal health coverage.

Second, the allocation of resources to treat and prevent measles should consider urban and rural communities. Measles outbreaks took a heavy toll on rural areas, where most of the study caregivers resided (88% were rural residents). This said, most of the measles-related healthcare services were provided at tertiary and secondary-level hospitals in urban areas (Sylhet city corporation, 91%), with only a few cases seen at a hospital in rural areas (Maulvibazar, 5%). Rural residents incurred additional costs for transportation and meals to access healthcare, increasing their out-of-pocket expenditures substantially compared to those residing in urban areas.

Furthermore, these estimates also highlight the weight of time loss and the indirect cost due to productivity loss for both users of government facilities and private healthcare, averaging 64–85% of the total cost of the former and 55% of the latter. Caregivers must spend a significant amount of time away from work and from the household to access care for measles treatment, hindering the household’s financial security. Public hospitals in rural areas could take charge of those patients requiring hospitalization instead of referring them to the larger centralized hospitals in Sylhet city corporation, thus reducing the need for significant travel to access healthcare.

Few studies have conducted primary data collection to estimate the cost of measles [23]. We recently published estimates of the cost of measles in Uganda, using the same disease definition and costing approach, and compared them in Supplementary Material: Tables S3 and S4 [12]. Most caregivers also sought care in secondary and tertiary hospitals (91%), avoiding lower level, rural facilities. For hospitalized care in those facilities, the government’s average cost per episode ranged from $12 to $17: not statistically significantly from the estimates presented here ($18–$23, see Table 1). Capital, labor, and overhead costs were similar, while medication costs were significantly lower in Uganda as fewer medications were reportedly prescribed for measles (based on patient medical records). Caregiver costs were significantly lower in Uganda, averaging $22 and $35 in out-of-pocket payments, and $44 and $70 in total economic costs for a hospitalized case in public and private healthcare facilities, respectively (compared to $48, $83, and $131, $182, see Table 2). Fewer caregivers in Uganda paid for medical care, drawing the mean costs down significantly. Those who paid also spent less by a factor of 2 to 14, beyond adjusting for purchasing power. Caregivers in Uganda spent significantly less on medications and non-medical costs. While time loss overall was not significantly different between the two, its monetary valuation for Bangladesh was 3- to 6-fold that of Uganda. This combination of government and caregiver costs makes up for a more considerable societal cost per episode of measles for Bangladesh than Uganda [12]. There is no other study estimating the cost of measles in low-income settings [23].

Such high medical costs are not uncommon for severe conditions requiring hospitalization care in Bangladesh: Ashraf et al. (2010) estimated that the societal cost of hospitalized severe pneumonia could reach $178 in 2007 US dollars ($310 in 2018), excluding non-medical and indirect costs [24]. In contrast, Sarker et al. (2013) show lower out-of-pocket payments (medical and non-medical) for hospitalized diarrhea due to cholera in Dhaka for the caregivers at $7 in 2011 US dollars ($10 in 2018). The lower cost for caregivers highlighted in this study could be due to the share of the treatment costs taken by the healthcare provider (an icddr,b hospital), to the fact that diarrheal diseases are routinely treated (compared to an outbreak disease like measles), and to the shorter distances to travel to access healthcare [25]. Indirect costs remained a significant share of the total cost [25, 26].

These cost estimates for measles provide new insights to understand the burden this disease poses to the healthcare system and households in Bangladesh, both in financial and economic terms. However, without assessing the impact of sequelae on the child’s future health and of mortality to a household’s expected income, our estimates are likely conservative. The extent to which a measles infection impacts a child’s future health is yet to be fully understood. Clinical studies pre-post measles infection revealed that children who survived went on with a weakened immune system. Measles seems to be severely weakening the child’s immunity against other infectious diseases for which the child had previously received a vaccine [27]. An episode of measles could create the need to re-vaccinate affected children, with all the costs vaccination entails.

The study had several limitations linked to the definition of the disease: access to laboratory confirmation was limited, and the child’s vaccination status was difficult to assess. When asked whether the child had an immunization card, nearly all caregivers reported that their child had one but did not have it at the time of their interview. Since we asked to see immunization cards to record the date when vaccines were given, we could not check the vaccination status of the children treated for measles. Additionally, the study estimates are conservative as the analysis does not include the medium to long-term costs of measles-related sequelae.

## Conclusion

This study is one of the few studies that examined the economic burden of a disease with a vaccine with a longstanding presence in national EPI. Considering the high effectiveness of MCV, these societal cost estimates directly quantify the benefits of expanding the measles eradication strategy started in 2012. The government should continue to improve the vaccination coverage, particularly by consolidating routine immunization to bridge the gap between high and low performing divisions. It is an essential contribution to health systems strengthening.

## Supplementary Information

**Additional file 1.** Supplementary tables and figures featuring additional subgroup comparisons and costs in 2018 Bangladeshi Takas (BDT).

## Data Availability

The datasets supporting the conclusions of this article are available in the Harvard Data Verse repository: Ahmed, Sayem; de Broucker, Gatien; Hasan, Md. Zahid; Mehdi, Gazi Golam; Martin del Campo, Jorge; Constenla, Dagna; Patenaude, Bryan; Uddin, Md. Jasim, 2020, “Cost of measles in children under 5 in Bangladesh (2017-18)”, 10.7910/DVN/ZXZEUY, Harvard Dataverse, V2, UNF:6:gGKfNTwvPYwBxaK9xYHtdw== [fileUNF].

## Abbreviations

BP: Dr. Bryan Patenaude
DC: Dr. Dagna Constenla
GD: Mr. Gatien de Broucker
GM: Mr. Gazi Golam Mehdi
JM: Dr. Jorge Martin del Campo
JU: Dr. Md. Jasim Uddin
SA: Dr. Sayem Ahmed
WA: Mr. Md. Wazed Ali
ZH: Mr. Md. Zahid Hasan
ANOVA: Analysis of Variance
BDT: Bangladeshi Takas
DOVE: Decade Of Vaccine Economics project
EPI: Expanded Program for Immunization
GDP: Gross Domestic Product
GHCC: Global Health Cost Consortium
GSK: GlaxoSmithKline
icddr,b: International Centre for Diarrhoeal Disease Research, Bangladesh
IRB: Institutional Review Board
MCV: Measles-Containing Vaccine
MCV1: First dose of Measles-Containing Vaccine
MCV2: Second dose of Measles-Containing Vaccine
PCA: Principal Component Analysis
PFP: Private For-Profit sector
PNFP: Private Not-For-Profit sector
USD: United States Dollars
