# Supplementary material for: The economic burden of measles in children under five in Bangladesh
Source: BMC Health Serv Res. 2020 Nov 10;20:1026. doi: 10.1186/s12913-020-05880-5 (PMC7653835; doi:10.1186/s12913-020-05880-5)
Supplement: Supplementary file 1 — Additional file 1. Supplementary tables and figures featuring additional subgroup comparisons and costs in 2018 Bangladeshi Takas (BDT). [file 12913_2020_5880_MOESM1_ESM.docx]

**Supplementary tables and figures in 2018 US dollars ($)**

***Table S1: Types of costs by perspective***

|  |  |  |  | | | |  |
| --- | --- | --- | --- | --- | --- | --- | --- |
| **Total cost from the societal perspective** | **Total cost from the household perspective**  (Caregiver) |  | **Total economic cost for the caregiver** | | | | |
|  |  |  | **Total direct (financial) cost for the caregiver** | |  |  |  |
|  |  |  | **Direct costs** | |  |  | **Indirect costs** |
|  |  |  | **Medical costs:** | **Non-medical costs:** |  |  |  |
|  |  |  | - Consultation or registration fees - Cost of investigations/diagnosis (radiology, laboratory tests) - Cost of hospitalization/bed for the patient - Cost of medications - Cost of medical supplies | - Cost of transportation to and from the healthcare facilities for the patient and the caregiver - Cost of meals for the patient and the caregiver during hospitalization - Miscellaneous costs (telephone calls, gifts from visitors) |  |  | - Productivity loss for the caregiver related to the acute episode of illness based on the time spent in the healthcare system |
|  |  |  |  |  |  |  |  |
|  | **Total cost from the healthcare provider perspective** (Government) |  | **Service costs:** | | | |  |
|  |  |  | - Capital costs (infrastructure, furniture, vehicles, laboratory and radiology instruments) - Cost of personnel (full-time, part-time and on-call staff) - Cost of overhead (building maintenance, utilities) - Cost of hospitalization/bed - Cost of medications - Cost of medical supplies | | | |  |
|  |  |  |  | | | |  |

***Table S2: Differences in caregiver costs in 2018 US dollars ($)***

| **Characteristic** | **n** | **Direct costs** | | | **Indirect costs** | | | **Total costs** | | |
| --- | --- | --- | --- | --- | --- | --- | --- | --- | --- | --- |
|  |  | Mean | SE | p-value | Mean | SE | p-value | Mean | SE | p-value |
| **Overall** | **95** | **$53.84** | **$3.17** | **-** | **$84.20** | **$7.10** | **-** | **$138.05** | **$9.34** | **-** |
| **Age group** |  |  |  |  |  |  |  |  |  |  |
| < 6 months | 16 | $42.69 | $4.95 | 0.143 | $80.10 | $9.38 | 0.108 | $122.79 | $11.29 | 0.250 |
| 6-11 months | 39 | $62.07 | $5.62 |  | $101.09 | $14.17 |  | $163.15 | $18.18 |  |
| 12-24 months | 19 | $51.15 | $7.09 |  | $60.63 | $10.69 |  | $111.78 | $15.71 |  |
| > 24 months | 21 | $49.49 | $5.90 |  | $77.31 | $12.93 |  | $126.79 | $17.59 |  |
| **Gender (child)** |  |  |  |  |  |  |  |  |  |  |
| Female | 39 | $50.91 | $5.21 | 0.442 | $68.60 | $7.50 | 0.120 | $119.51 | $11.98 | 0.098 |
| Male | 56 | $55.88 | $3.98 |  | $95.07 | $10.68 |  | $150.96 | $13.28 |  |
| **Gender (caregiver)** |  |  |  |  |  |  |  |  |  |  |
| Female | 78 | $51.31 | $3.10 | 0.298 | $87.41 | $8.12 | 0.337 | $138.72 | $10.31 | 0.879 |
| Male | 17 | $65.47 | $10.35 |  | $69.51 | $13.57 |  | $134.97 | $22.72 |  |
| **Study area** |  |  |  |  |  |  |  |  |  |  |
| City corp. | 86 | $56.25 | $3.23 | 0.003 | $85.05 | $7.45 | 0.715 | $141.29 | $9.71 | 0.284 |
| *Sylhet* | 86 | $56.25 | $3.23 | *-* | $85.05 | $7.45 | *-* | $141.29 | $9.71 | *-* |
| *Rajshahi* | 0 | - | - |  | - | - |  | - | - |  |
| Rural district | 9 | $30.83 | $10.34 |  | $76.13 | $24.95 |  | $106.96 | $33.35 |  |
| *Maulvibazar* | 5 | $53.46 | $9.76 | *0.139* | $126.06 | $27.94 | *0.049* | $179.52 | $31.20 | *0.014* |
| *Natore*^[A]^ | 4 | $2.51 | $1.44 |  | $13.74 | $9.88 |  | $16.25 | $10.68 |  |
| **Residence** |  |  |  |  |  |  |  |  |  |  |
| Rural | 84 | $56.18 | $3.28 | 0.040 | $86.65 | $7.92 | 0.524 | $142.84 | $10.20 | 0.158 |
| Urban | 11 | $35.96 | $9.74 |  | $65.51 | $9.35 |  | $101.47 | $18.04 |  |
| **Type of visit** |  |  |  |  |  |  |  |  |  |  |
| Inpatient | 91 | $56.10 | $3.10 | 0.000 | $87.31 | $7.23 | 0.000 | $143.40 | $9.34 | 0.000 |
| Outpatient | 4 | $2.51 | $1.44 |  | $13.74 | $9.88 |  | $16.25 | $10.68 |  |
| **Facilities** |  |  |  |  |  |  |  |  |  |  |
| Public | 73 | $45.17 | $2.77 | 0.000 | $79.57 | $8.06 | 0.237 | $124.74 | $10.01 | 0.009 |
| PNFP | 22 | $82.61 | $7.45 |  | $99.58 | $14.96 |  | $182.20 | $20.59 |  |
| **Length of stay (IPD)^[B]^** |  |  |  |  |  |  |  |  |  |  |
| < 5 days | 24 | $44.34 | $5.10 | 0.022 | $54.08 | $7.51 | 0.000 | $98.42 | $10.36 | 0.000 |
| >= 5 days | 67 | $60.31 | $3.68 |  | $99.20 | $9.05 |  | $159.51 | $11.56 |  |
| **Asset quintiles^[C]^** |  |  |  |  |  |  |  |  |  |  |
| Poorest | 27 | $50.31 | $3.23 | 0.099 | $67.54 | $7.13 | 0.001 | $117.86 | $8.83 | 0.006 |
| 2nd | 11 | $33.45 | $9.65 |  | $38.55 | $11.04 |  | $72.00 | $19.11 |  |
| 3rd | 20 | $54.74 | $5.04 |  | $69.96 | $6.48 |  | $124.69 | $8.44 |  |
| 4th | 18 | $63.76 | $6.50 |  | $96.26 | $16.49 |  | $160.02 | $21.04 |  |
| Richest | 19 | $60.32 | $11.05 |  | $137.87 | $24.84 |  | $198.19 | $33.40 |  |

Legend: SE, Standard Error; n, number of caregivers.
^[A]^ All cases in Natore district (Rajshahi division) were outpatient cases.
^[B]^ Length of stay includes only hospitalized cases of measles (n = 91).
^[C]^ The 2^nd^ quintile (n = 11) contains all 4 outpatient cases, hence lower costs.

**Supplementary analysis: Comparison of healthcare costs between Bangladesh and Uganda**

All costs are presented in 2018 US dollars, converted at the following exchange rate: $1 = BDT 83.5; $1 = UGX 3,727.

We estimated the economic burden of measles in Bangladesh (present paper) and Uganda (reference below), using the same disease definition and costing approach, and conducting data collection concurrently. In Tables S3 and S4, we compare the cost estimates for Bangladesh and Uganda for hospitalized measles. A ratio of the mean costs of Bangladesh over those of Uganda is calculated in the last column, with productivity loss adjusted for time loss ([ratio]).

Transportation costs before the current visit were not assessed in Bangladesh.

Reference: De Broucker G, Ssebagereka A, Apolot RR, Aloysius M, Ekirapa Kiracho E, Patenaude B, Constenla D: **The economic burden of measles in children under five in Uganda**. *Vaccine: X* 2020, **6**:100077.

***Table S3: Household costs for a hospitalized episode of measles in 2018 US dollars ($)***

| **Timing** | **Type** | **Cost** | **Public healthcare facilities** | | | | | | | | **Mean cost ratio: Bangladesh / Uganda** |
| --- | --- | --- | --- | --- | --- | --- | --- | --- | --- | --- | --- |
|  |  |  | **Bangladesh (n=69)** | | | | **Uganda (n=84)** | | | |  |
|  |  |  | Mean | 95% CI | | %(c>0) | Mean | 95% CI | | %(c>0) |  |
| **Before current visit^[A]^** | **Direct medical costs** | Consultation | $1.11 | $0.42 | $1.82 | 30% | $0.14 | -$0.07 | $0.36 | 4% | 7.9 |
|  |  | Investigations | $0.50 | -$0.19 | $1.20 | 4% | $0.00 | $0.00 | $0.00 | 0% | . |
|  |  | Medications | $1.92 | $1.03 | $2.79 | 39% | $1.08 | -$0.04 | $2.20 | 13% | 1.8 |
|  |  | Hospitalization | $0.00 | $0.00 | $0.00 | 0% | $0.00 | $0.00 | $0.00 | 0% | . |
|  |  | **Total** | **$3.53** | **$1.90** | **$5.16** | **45%** | **$1.22** | **$0.09** | **$2.36** | **17%** | **2.9** |
|  | **Direct non-medical costs** | Transportation | *Not included* | | | | $0.26 | $0.13 | $0.39 | 21 |  |
|  |  | Meals | $0.18 | -$0.02 | $0.38 | 6% | $0.21 | $0.00 | $0.42 | 8% | 0.9 |
|  |  | Other | $0.18 | -$0.02 | $0.40 | 4% | $0.20 | $0.05 | $0.35 | 11% | 0.9 |
|  |  | **Total** | **$0.36** | **-$0.02** | **$0.75** | **6%** | **$0.66** | **$0.29** | **$1.04** | **25%** | **0.5** |
|  | **Indirect costs** | Time loss [days] | 1.54 | 1.25 | 1.84 | 100% | 0.34 | 0.14 | 0.54 | 37% |  |
|  |  | **Productivity loss** | **$28.85** | **$21.58** | **$36.12** | **100%** | **$0.99** | **$0.35** | **$1.63** | **36%** | **[6.4]** |
| **Current** | **Direct medical costs** | Consultation | $0.30 | $0.29 | $0.31 | 100% | $0.00 | $0.00 | $0.00 | 0% | . |
|  |  | Investigations | $4.74 | $3.31 | $6.18 | 55% | $0.08 | -$0.05 | $0.21 | 4% | 59.3 |
|  |  | Medications | $9.60 | $7.44 | $11.77 | 97% | $3.73 | $2.73 | $4.72 | 68% | 2.6 |
|  |  | Hospitalization | $0.00 | $0.00 | $0.00 | 0% | $0.00 | $0.00 | $0.00 | 0% | . |
|  |  | **Total** | **$14.65** | **$12.19** | **$17.09** | **100%** | **$3.81** | **$2.81** | **$4.82** | **68%** | **3.8** |
|  | **Direct non-medical costs** | Transportation | $11.29 | $9.08 | $13.52 | 100% | $1.62 | $1.21 | $2.03 | 96% | 7.0 |
|  |  | Meals | $10.79 | $9.09 | $12.50 | 94% | $4.97 | $4.13 | $5.81 | 94% | 2.2 |
|  |  | Other | $3.90 | $3.40 | $4.40 | 97% | $1.33 | $1.00 | $1.65 | 74% | 2.9 |
|  |  | **Total** | **$26.00** | **$22.69** | **$29.29** | **100%** | **$7.92** | **$6.79** | **$9.05** | **100%** | **3.3** |
|  | **Indirect costs** | Time loss [days] | 2.88 | 2.54 | 3.21 | 100% | 3.3 | 2.87 | 3.74 | 99% |  |
|  |  | **Productivity loss** | **$54.51** | **$44.23** | **$64.80** | **100%** | **$14.14** | **$9.42** | **$18.85** | **95%** | **[4.4]** |
| **Follow-up^[A]^** | **Direct medical costs** | Consultation | $0.02 | -$0.01 | $0.06 | 3% | $0.00 | $0.00 | $0.00 | 0% | . |
|  |  | Investigations | $0.26 | -$0.26 | $0.78 | 1% | $0.00 | $0.00 | $0.00 | 0% | . |
|  |  | Medications | $2.29 | $1.86 | $2.72 | 75% | $2.25 | $1.30 | $3.20 | 33% | 1.0 |
|  |  | Hospitalization | $0.00 | $0.00 | $0.00 | 0% | $0.00 | $0.00 | $0.00 | 0% | . |
|  |  | **Total** | **$2.57** | **$1.88** | **$3.27** | **75%** | **$2.25** | **$1.30** | **$3.20** | **33%** | **1.1** |
|  | **Direct non-medical costs** | Transportation | $0.29 | -$0.26 | $0.85 | 3% | $0.68 | $0.39 | $0.97 | 43% | 0.4 |
|  |  | Meals | $0.24 | -$0.24 | $0.73 | 1% | $4.72 | $3.46 | $5.99 | 52% | 0.1 |
|  |  | Other | $0.00 | $0.00 | $0.00 | 0% | $1.75 | $1.20 | $2.30 | 48% | 0.0 |
|  |  | **Total** | **$0.54** | **-$0.50** | **$1.57** | **3%** | **$7.15** | **$5.43** | **$8.87** | **57%** | **0.1** |
|  | **Indirect costs** | Time loss [days] | 0.04 | -0.04 | 0.11 | 3% | 2.94 | 2.1 | 3.77 | 46% |  |
|  |  | **Productivity loss** | **$0.44** | **-$0.43** | **$1.31** | **3%** | **$9.31** | **$5.67** | **$12.95** | **45%** | **[3.5]** |
| **Total direct (financial) cost** | | | **$47.64** | **$42.38** | **$52.90** | **100%** | **$21.79** | **$18.91** | **$24.67** | **100%** | **2.2** |
| **Total economic cost** | | | **$131.03** | **$110.93** | **$151.13** | **100%** | **$44.25** | **$36.98** | **$51.51** | **100%** | **3.0** |

Legend: SD, Standard Deviation; %(c>0), proportion of caregivers with a cost/time spent valued over zero.
^[A]^ Includes costs incurred at public and private healthcare facilities and providers.

***Table S4: Household costs for a hospitalized episode of measles in 2018 US dollars ($)***

| **Timing** | **Type** | **Cost** | **Private not-for-profit healthcare facilities** | | | | | | | | **Cost ratio: Bangladesh / Uganda** |
| --- | --- | --- | --- | --- | --- | --- | --- | --- | --- | --- | --- |
|  |  |  | **Bangladesh (n=22)** | | | | **Uganda (n=32)** | | | |  |
|  |  |  | Mean | 95% CI | | %(c>0) | Mean | 95% CI | | %(c>0) |  |
| **Before current visit^[A]^** | **Direct medical costs** | Consultation | $1.53 | $0.40 | $2.67 | 45% | $0.17 | -$0.17 | $0.51 | 3% | 9.0 |
|  |  | Investigations | $0.02 | -$0.02 | $0.08 | 5% | $0.03 | -$0.03 | $0.08 | 3% | 0.7 |
|  |  | Medications | $5.62 | $2.85 | $8.37 | 77% | $0.33 | -$0.04 | $0.70 | 13% | 17.0 |
|  |  | Hospitalization | $0.00 | $0.00 | $0.00 | 0% | $0.00 | $0.00 | $0.00 | 0% | . |
|  |  | **Total** | **$7.17** | **$4.18** | **$10.16** | **77%** | **$0.52** | **$0.04** | **$1.01** | **19%** | **13.8** |
|  | **Direct non-medical costs** | Transportation | *Not included* | | | | $0.37 | -$0.16 | $0.91 | 5 |  |
|  |  | Meals | $0.92 | -$0.25 | $2.11 | 18% | $0.22 | -$0.08 | $0.51 | 9% | 4.2 |
|  |  | Other | $0.60 | -$0.11 | $1.31 | 18% | $0.25 | -$0.01 | $0.51 | 13% | 2.4 |
|  |  | **Total** | **$1.52** | **$0.00** | **$3.04** | **23%** | **$0.84** | **$0.02** | **$1.66** | **22%** | **1.8** |
|  | **Indirect costs** | Time loss [days] | 1.13 | 0.84 | 1.42 | 100% | 0.45 | 0.12 | 0.79 | 28% |  |
|  |  | **Productivity loss** | **$24.72** | **$14.51** | **$34.92** | **100%** | **$3.01** | **-$0.24** | **$6.26** | **28%** | **[3.3]** |
| **Current** | **Direct medical costs** | Consultation | $0.84 | $0.71 | $0.98 | 100% | $8.43 | $6.15 | $10.70 | 72% | 0.1 |
|  |  | Investigations | $0.81 | -$0.56 | $2.20 | 9% | $1.07 | -$0.19 | $2.33 | 16% | 0.8 |
|  |  | Medications | $24.54 | $16.98 | $32.08 | 100% | $2.82 | -$1.11 | $6.75 | 9% | 8.7 |
|  |  | Hospitalization | $16.23 | $9.72 | $22.72 | 100% | $1.13 | -$1.09 | $3.35 | 6% | 14.4 |
|  |  | **Total** | **$42.42** | **$33.03** | **$51.81** | **100%** | **$13.45** | **$8.73** | **$18.17** | **81%** | **3.2** |
|  | **Direct non-medical costs** | Transportation | $10.06 | $5.32 | $14.80 | 100% | $2.48 | $1.25 | $3.71 | 91% | 4.1 |
|  |  | Meals | $10.46 | $8.81 | $12.10 | 100% | $1.91 | $0.53 | $3.29 | 25% | 5.5 |
|  |  | Other | $7.81 | $5.16 | $10.47 | 100% | $2.79 | $1.64 | $3.94 | 66% | 2.8 |
|  |  | **Total** | **$28.32** | **$21.78** | **$34.86** | **100%** | **$7.19** | **$4.53** | **$9.84** | **97%** | **3.9** |
|  | **Indirect costs** | Time loss [days] | 3.25 | 2.84 | 3.66 | 100% | 3.98 | 2.88 | 5.08 | 100% |  |
|  |  | **Productivity loss** | **$74.86** | **$50.37** | **$99.37** | **100%** | **$17.78** | **$8.06** | **$27.51** | **94%** | **[5.2]** |
| **Follow-up^[A]^** | **Direct medical costs** | Consultation | $0.00 | $0.00 | $0.00 | 0% | $9.34 | $6.77 | $11.91 | 56% | 0.0 |
|  |  | Investigations | $0.00 | $0.00 | $0.00 | 0% | $0.00 | $0.00 | $0.00 | 0% | . |
|  |  | Medications | $3.07 | $1.75 | $4.40 | 73% | $0.00 | $0.00 | $0.00 | 0% | . |
|  |  | Hospitalization | $0.00 | $0.00 | $0.00 | 0% | $0.00 | $0.00 | $0.00 | 0% | . |
|  |  | **Total** | **$3.07** | **$1.75** | **$4.40** | **73%** | **$9.34** | **$6.77** | **$11.91** | **56%** | **0.3** |
|  | **Direct non-medical costs** | Transportation | $0.00 | $0.00 | $0.00 | 0% | $1.46 | $0.88 | $2.03 | 63% | 0.0 |
|  |  | Meals | $0.00 | $0.00 | $0.00 | 0% | $0.46 | -$0.20 | $1.13 | 6% | 0.0 |
|  |  | Other | $0.11 | -$0.02 | $0.24 | 14% | $3.66 | $2.25 | $5.07 | 56% | 0.0 |
|  |  | **Total** | **$0.11** | **-$0.02** | **$0.24** | **14%** | **$5.58** | **$3.85** | **$7.31** | **66%** | **0.0** |
|  | **Indirect costs** | Time loss [days] | 0 | 0 | 0 | 0% | 5.07 | 3.69 | 6.46 | 66% |  |
|  |  | **Productivity loss** | **$0.00** | **$0.00** | **$0.00** | **0%** | **$13.99** | **$8.46** | **$19.52** | **63%** | **[.]** |
| **Total direct (financial) cost** | | | **$82.61** | **$67.13** | **$98.10** | **100%** | **$34.13** | **$28.17** | **$40.08** | **100%** | **2.4** |
| **Total economic cost** | | | **$182.20** | **$139.38** | **$225.02** | **100%** | **$65.20** | **$53.49** | **$76.90** | **100%** | **2.8** |

Legend: SD, Standard Deviation; %(c>0), proportion of caregivers with a cost/time spent valued over zero.
^[A]^ Includes costs incurred at public and private healthcare facilities and providers.

**Supplementary tables and figures in 2018 Bangladeshi Takas (BDT)**

***Table S5: Government costs for an episode of measles in 2018 BDT***

| **Level, name & type of care** | n | **Service costs** | | | | | |
| --- | --- | --- | --- | --- | --- | --- | --- |
|  |  | Capital | Overhead | Labor | Supplies | Medications | **Total** |
| **Primary level** |  |  |  |  |  |  |  |
| Bashbaria Community Clinic |  |  |  |  |  |  |  |
| *Outpatient care* | 1 | 38.21 | 0.28 | 45.40 | 0 | 0 | **83.89** |
| Baraigram Upazila Health Complex |  |  |  |  |  |  |  |
| *Outpatient care* | 2 | 6.97 | 10.72 | 82.76 | 1.34 | 41.00 | **142.79** |
| Zoary Union Sub Center |  |  |  |  |  |  |  |
| *Outpatient care* | 1 | 19.03 | 0.21 | 100.02 | 0 | 0 | **119.26** |
|  |  |  |  |  |  |  |  |
| **Secondary level** |  |  |  |  |  |  |  |
| Moulvibazar District Hospital, Moulvibazar |  |  |  |  |  |  |  |
| *Inpatient care* | 5 | 26.44 | 81.64 | 480.73 | 17.63 | 859.92 | **1466.36** |
|  |  |  |  |  |  |  |  |
| **Tertiary level** |  |  |  |  |  |  |  |
| Sylhet M. A. G. Osmani Medical College Hospital |  |  |  |  |  |  |  |
| *Inpatient care* | 64 | 110.09 | 67.83 | 858.85 | 77.19 | 796.15 | **1910.11** |

Legend: n, number of caregivers interviewed at the facility.

***Table S6: Total caregiver costs for a hospitalized episode of measles in 2018 BDT***

| **INPATIENT VISIT** | | | | | | | | | | | |
| --- | --- | --- | --- | --- | --- | --- | --- | --- | --- | --- | --- |
| **Timing** | **Cost** | **Public healthcare facilities (n=69)** | | | | | **Private not-for-profit healthcare facilities (n=22)** | | | | |
|  |  | Mean | SD | 95% CI | | n(c>0) | Mean | SD | 95% CI | | n(c>0) |
| **Before current visit^[A]^** | Direct medical | 295 | 566 | 159 | 431 | 31 | 599 | 563 | 349 | 848 | 17 |
|  | Direct non-medical | 30 | 134 | -2 | 63 | 4 | 127 | 286 | 0 | 254 | 5 |
|  | Indirect | 2409 | 2507 | 1802 | 3016 | 69 | 2064 | 1922 | 1212 | 2916 | 22 |
|  | *Time loss [days]* | 1.54 | 1.24 | 1.25 | 1.84 | 69 | 1.13 | 0.67 | 0.84 | 1.42 | 22 |
| **Current visit** | Direct medical | 1223 | 851 | 1018 | 1427 | 69 | 3542 | 1768 | 2758 | 4326 | 22 |
|  | Direct non-medical | 2171 | 1145 | 1895 | 2446 | 69 | 2365 | 1231 | 1819 | 2911 | 22 |
|  | Indirect | 4552 | 3575 | 3693 | 5411 | 69 | 6251 | 4613 | 4206 | 8297 | 22 |
|  | *Time loss [days]* | 2.88 | 1.41 | 2.54 | 3.21 | 69 | 3.25 | 0.93 | 2.84 | 3.66 | 22 |
| **Follow-up^[A]^** | Direct medical | 215 | 242 | 157 | 273 | 52 | 256 | 250 | 146 | 367 | 16 |
|  | Direct non-medical | 45 | 361 | -42 | 131 | 2 | 9 | 25 | -2 | 20 | 3 |
|  | Indirect | 37 | 301 | -36 | 109 | 2 | 0 | 0 | 0 | 0 | 0 |
|  | *Time loss [days]* | 0.04 | 0.30 | -0.04 | 0.11 | 2 | 0.00 | 0.00 | 0.00 | 0.00 | 0 |
| **Total direct (financial) cost** | | **3978** | **1826** | **3735** | **4628** | **69** | **6898** | **2916** | **5847** | **8460** | **22** |
| **Total economic cost** | | **10941** | **6985** | **9263** | **12619** | **69** | **15214** | **8065** | **11638** | **18789** | **22** |
| **OUTPATIENT VISIT** | | | | | | | | | | | |
| **Timing** | **Cost** | **Public healthcare facilities (n=4)** | | | | |  |  |  |  |  |
|  |  | Mean | SD | 95% CI | | n(c>0) |  |  |  |  |  |
| **Before current visit^[A]^** | Direct medical | 0 | 0 | 0 | 0 | 0 |  |  |  |  |  |
|  | Direct non-medical | 0 | 0 | 0 | 0 | 0 |  |  |  |  |  |
|  | Indirect | 24 | 26 | -17 | 65 | 4 |  |  |  |  |  |
|  | *Time loss [days]* | 0.02 | 0.03 | -0.02 | 0.07 | 4 |  |  |  |  |  |
| **Current visit** | Direct medical | 2 | 2 | 0 | 3 | 3 |  |  |  |  |  |
|  | Direct non-medical | 5 | 5 | -11 | 21 | 1 |  |  |  |  |  |
|  | Indirect | 1112 | 1632 | -1486 | 3709 | 4 |  |  |  |  |  |
|  | *Time loss [days]* | 1.10 | 1.64 | -1.51 | 3.71 | 4 |  |  |  |  |  |
| **Follow-up^[A]^** | Direct medical | 174 | 202 | -148 | 495 | 2 |  |  |  |  |  |
|  | Direct non-medical | 30 | 60 | -65 | 125 | 1 |  |  |  |  |  |
|  | Indirect | 11 | 22 | -24 | 46 | 1 |  |  |  |  |  |
|  | *Time loss [days]* | 0.01 | 0.02 | -0.02 | 0.04 | 1 |  |  |  |  |  |
| **Total direct (financial) cost** | | **210** | **241** | **-302** | **1020** | **4** |  |  |  |  |  |
| **Total economic cost** | | **1357** | **1784** | **-1482** | **4196** | **4** |  |  |  |  |  |

Legend: SD, Standard Deviation; n(c>0), number of caregivers with a cost/time spent valued over zero.
^[A]^ Includes costs incurred at public and private healthcare facilities and providers.

***Table S7: Differences in caregiver costs in 2018 BDT***

| **Characteristic** | **n** | **Direct costs** | | | **Indirect costs** | | | **Total costs** | | |
| --- | --- | --- | --- | --- | --- | --- | --- | --- | --- | --- |
|  |  | Mean | SE | p-value | Mean | SE | p-value | Mean | SE | p-value |
| **Overall** | **95** | **4496** | **265** | **-** | **7031** | **593** | **-** | **11527** | **780** | **-** |
| **Age group** |  |  |  |  |  |  |  |  |  |  |
| < 6 months | 16 | 3565 | 413 | 0.143 | 6688 | 783 | 0.108^[B]^ | 10253 | 943 | 0.250^[B]^ |
| 6-11 months | 39 | 5183 | 469 |  | 8441 | 1183 |  | 13623 | 1518 |  |
| 12-24 months | 19 | 4271 | 592 |  | 5063 | 893 |  | 9334 | 1312 |  |
| > 24 months | 21 | 4132 | 493 |  | 6455 | 1080 |  | 10587 | 1469 |  |
| **Gender (child)** |  |  |  |  |  |  |  |  |  |  |
| Female | 39 | 4251 | 435 | 0.442 | 5728 | 626 | 0.120^[B]^ | 9979 | 1000 | 0.098^[B]^ |
| Male | 56 | 4666 | 332 |  | 7938 | 892 |  | 12605 | 1109 |  |
| **Gender (caregiver)** |  |  |  |  |  |  |  |  |  |  |
| Female | 78 | 4284 | 259 | 0.298^[B]^ | 7299 | 678 | 0.337 | 11583 | 861 | 0.879^[B]^ |
| Male | 17 | 5467 | 864 |  | 5804 | 1133 |  | 11270 | 1897 |  |
| **Study area** |  |  |  |  |  |  |  |  |  |  |
| City corp. | 86 | 4697 | 270 | 0.003^[B]^ | 7102 | 622 | 0.715 | 11798 | 811 | 0.284^[B]^ |
| *Sylhet* | 86 | 4697 | 270 | *-* | 7102 | 622 | *-* | 11798 | 811 | *-* |
| *Rajshahi* | 0 | - | - |  | - | - |  | - | - |  |
| Rural district | 9 | 2574 | 863 |  | 6357 | 2083 |  | 8931 | 2785 |  |
| *Maulvibazar* | 5 | 4464 | 815 | *0.139*^[B]^ | 10526 | 2333 | *0.049* | 14990 | 2605 | *0.014*^[B]^ |
| *Natore*^[C]^ | 4 | 210 | 120 |  | 1147 | 825 |  | 1357 | 892 |  |
| **Residence** |  |  |  |  |  |  |  |  |  |  |
| Rural | 84 | 4691 | 274 | 0.040 | 7235 | 661 | 0.524^[B]^ | 11927 | 852 | 0.158 |
| Urban | 11 | 3003 | 813 |  | 5470 | 781 |  | 8473 | 1506 |  |
| **Type of visit** |  |  |  |  |  |  |  |  |  |  |
| Inpatient | 91 | 4684 | 259 | 0.000^[B]^ | 7290 | 604 | 0.000^[B]^ | 11974 | 780 | 0.000 |
| Outpatient | 4 | 210 | 120 |  | 1147 | 825 |  | 1357 | 892 |  |
| **Facilities** |  |  |  |  |  |  |  |  |  |  |
| Public | 73 | 3772 | 231 | 0.000^[B]^ | 6644 | 673 | 0.237^[B]^ | 10416 | 836 | 0.009 |
| PNFP | 22 | 6898 | 622 |  | 8315 | 1249 |  | 15214 | 1719 |  |
| **Length of stay (IPD)^[A]^** |  |  |  |  |  |  |  |  |  |  |
| < 5 days | 24 | 3702 | 426 | 0.022 | 4516 | 627 | 0.000^[B]^ | 8218 | 865 | 0.000^[B]^ |
| >= 5 days | 67 | 5036 | 307 |  | 8283 | 756 |  | 13319 | 965 |  |
| **Asset quintiles^[D]^** |  |  |  |  |  |  |  |  |  |  |
| Poorest | 27 | 4201 | 270 | 0.099^[B]^ | 5640 | 595 | 0.001^[B]^ | 9841 | 737 | 0.006^[B]^ |
| 2nd | 11 | 2793 | 806 |  | 3219 | 922 |  | 6012 | 1596 |  |
| 3rd | 20 | 4571 | 421 |  | 5842 | 541 |  | 10412 | 705 |  |
| 4th | 18 | 5324 | 543 |  | 8038 | 1377 |  | 13362 | 1757 |  |
| Richest | 19 | 5037 | 923 |  | 11512 | 2074 |  | 16549 | 2789 |  |

Legend: SE, Standard Error; n, number of caregivers.
^[A]^ All cases in Natore district (Rajshahi division) were outpatient cases.
^[B]^ Length of stay includes only hospitalized cases of measles (n = 91).
^[C]^ The 2^nd^ quintile (n = 11) contains all 4 outpatient cases, hence lower costs.

***Figure S1: Societal costs for measles in Bangladesh in 2018 BDT***
